# Toward Union

**Published:** 1918-03-23

**Authors:** 


### NURSING AFFAIRS IN IRELAND.

### TOWARD UNION.

Concerning union there is a great deal to be said and thought. If there is union, nothing else matters. If there is no union, Acts of Parliament are powerless to effect reform. British nurses ought to think this statement over, argue it out together, and seek to discover if it contains a flaw. Never mind about laymen, or limited companies, or democracy. Let there be one idea paramount—a Union of Trained Nurses, formed for their material benefit, and for nothing else—a Union which will ramify to the remotest bounds of the Empire. This is an ideal easily possible of attainment. Why should not nurses, like doctors, printers, engineers, and other workers, unite for their common and individual benefit and protection? It is not a matter of this society or that, but of articulating with one voice, harmonious and triumphant. Nothing can be more damaging, depressing, and discordant than the thousand unblended notes that fill the air. Nurses are members of one great Order, irrespective of race, religion, or politics. They are all engaged at the same splendid duty, and their interests are as much in common and as pressing as those of hungry men. Their needs with respect to hours of employment, remuneration, holidays, and pension are identical, and these can never be satisfied unless they gather together in one camp.

British nurses at the moment have the public ear. They have served their country, and the country will stand by them. And what I desire most of all to impress on the rank and file is that in such a movement faction-mongers are helpless. They are potent to hinder legislation, especially during the war, for in the most favourable circumstances the passing of an Act of Parliament would at present be extremely difficult. Personally, as I have more than once stated, I attach very small importance to an Act of Parliament establishing State Registration, but I attach infinite importance to the forming of a Union of Nurses.

The College of Nursing is an institution magnificently conceived and full of potentiality. Its basic principle is union for the betterment of the nurse, and its dominant personalities are men and women whose continued office depends on the votes of the members, which is the final answer to all criticism. All the talk about "gold-bugs," "employers," and "plutocrats" is nothing but empty sound. What would it profit any of these people individually, or all of them collectively, to keep nurses in subjection? When a man commits a murder he has a motive; when the masters of the slave traffic fought for their rights they had a motive. Will any hostile critic state in plain terms the possible underlying evil motive of public men and women of influence who have taken up the cause of British nurses? Let us have it out, and let all the cards be on view, or are they too proud or too cunning to reply? I have no sympathy with innuendo: it is merely a cover for dishonesty of purpose.

It is for the rank and file of the profession to be wise in time. Do not miss the tide: it is the nurses' hour, and we know not what the morrow will bring. A Union of Nurses is a bread-and-butter business from beginning to end, and when it is formed the little thrones on which the leaders of faction are perched will become dwarfed into wooden stools, a fact which in their inmost souls they realise and fear.

